# Limited Durability of Improvements in Infection Prevention and Control Practices Following Reactive Interventions Leaves Healthcare Facilities Vulnerable to Ebola Virus Transmission

**DOI:** 10.1093/cid/ciag192

**Published:** 2026-03-18

**Authors:** Joy Yang, Kasereka Masumbuko Claude, Emily Kimani, Michael T Hawkes

**Affiliations:** Department of Pediatrics, University of British Columbia, Vancouver, BC, Canada; Department of Medicine, Université Catholique du Graben, Butembo, Democratic Republic of the Congo; Department of Biological Sciences, University of Alberta, Edmonton, AB, Canada; Department of Pediatrics, University of British Columbia, Vancouver, BC, Canada

**Keywords:** Ebola virus disease, infection prevention and control, Africa, epidemic, preparedness

## Abstract

We assessed the impact and durability of an infection prevention and control (IPC) bundle intervention during the Kivu/Ituri Ebola virus outbreak (2018–2020). IPC scores increased, then declined 6 months postintervention (median 19/36, 30/36, and 28/36, *P* < .0001). Without sustained IPC, health facilities remain vulnerable to nosocomial transmission in future outbreaks.

On 1 September 2025, the Democratic Republic of Congo (DRC) declared its 16th Ebola virus disease (EVD) outbreak [1]. As of the discharge of the last EVD patient on October 19, there were a total of 64 confirmed or probable cases and 45 deaths (67% case fatality rate) [2]. Alarmingly, 4 healthcare workers were among the fatalities, pointing to nosocomial transmission of EVD and inadequate infection prevention and control (IPC) practices [3]. Despite extensive national experience in implementing IPC measures during 15 previous EVD outbreaks, the latest 2025 epidemic raised questions about their effectiveness and durability.

Healthcare facilities can serve as amplification points during EVD outbreaks [4, 5]. EVD is transmitted through direct contact with blood or body fluids [6]. Because early symptoms of EVD are nonspecific, it is often mistaken for other infectious diseases such as malaria, yellow fever, or dysentery [6]. Unless IPC measures are consistently applied in areas at risk of EVD outbreaks, nosocomial transmission within healthcare facilities may occur before infectious patients are clinically recognized [7].

We analyzed the impact and durability of an IPC bundle intervention implemented during the country's 10th epidemic (2018–2020) and highlighted implications for preventing nosocomial EVD transmission.

## METHODS

### Study Design

We conducted a quasi-experimental (before–after) study to evaluate the impact and durability of an IPC bundle intervention. This study was conducted from June 2019 to May 2020 across 92 healthcare facilities in Butembo, the DRC. The study occurred during the 2018–2020 Kivu/Ituri EVD outbreak, in which over 3000 EVD cases and 2000 deaths were reported [8]. All healthcare facilities were private primary health centers representative of local healthcare settings.

### Infection Prevention and Control Bundle Intervention

The bundle intervention was supported by the WHO and involved IPC kit provision with healthcare worker capacity-building. Kit items included disposable gloves, masks, alcohol-based hand rub gel, needle disposal safety box, soap, chlorine, coveralls, face shields, aprons, goggles, rubber boots, and EVD case definition posters [9]. Consumables (eg, gloves and alcohol-based hand rub) were replenished monthly for 4 months.

### Training of Study Personnel

The IPC bundle implementation and evaluation were conducted by 45 final-year medical students at the Université Catholique du Graben (Butembo, the DRC). Prior to baseline data collection, the students underwent a 7-day “train-the-trainers” program developed and facilitated by the Ministry of Health of the DRC. The curriculum included (1) standard IPC precaution measures; (2) case detection and triage strategies; (3) suspected case isolation protocols; and (4) waste management. On-site IPC mentorship to healthcare workers was provided by medical students, with each student overseeing an average of 4 healthcare facilities. Students were additionally trained in data collection using a standardized scorecard and were assigned to the same healthcare facilites throughout the study.

### Infection Prevention and Control Scorecard

A 36-item dichotomous (yes/no) standardized scorecard, developed based on WHO Ebola IPC assessment frameworks and adapted to the local DRC context, was used to evaluate compliance with IPC practices including personnel, isolation areas, triage practice, personal protective equipment (PPE) availability, and waste management (Supplementary Table 1). The scorecard was assessed at baseline, immediately postintervention, and at 6-month follow-up. The aggregate scores were calculated for each healthcare facility by summing individual scores from each item, with 36 representing full compliance.

### Statistical Analysis and Sample Size Calculation

Statistical analyses were performed using RStudio version 4.1 (RStudio, Inc., Boston, MA). Data were summarized as median and interquartile range (IQR). The Wilcoxon signed-rank test was performed to assess whether there was a difference in mean IPC scores between time points. The proportion of healthcare facilities compliant with each scorecard item was compared between timepoints using the McNemar test with Bonferroni correction for multiple (36) comparisons. A sample size of 54 facilities was required to detect a change of 2 points or more (on the 36-point scale), with 80% power at the α = .05 level of significance. This sample size calculation used the nonparametric Wilcoxon signed-rank test on simulated paired data and assumed that a mean (standard deviation) reduction of 2 (5) points would represent a clinically significant change.

## RESULTS

The intervention and evaluation occurred along the following timeline: (1) June 2019, baseline preintervention survey of health facilities; (2) June to September 2019, IPC bundle intervention; (3) October 2019, immediate postintervention survey; and (4) May 2020, follow-up survey for durability of intervention.

Ninety-two healthcare facilities were assessed at all 3 time points. The median number of beds per facility was 18 (IQR 10–35), and the median number of healthcare workers per facility was 9 (IQR 6–20). The baseline median IPC score was 19 (IQR 14–26). The median score increased to 30 (IQR 28–33) after the intervention (*P* < .0001). A statistically significant improvement was noted for 29 of 36 items (81%) on the scorecard (Supplementary Table 1). Several items that already had high levels of compliance before the intervention did not increase significantly, including waste incineration; presence of emergency contact (alert) number; response team alerted in case of EVD exposure; and ongoing training of healthcare workers in IPC (Supplementary Table 1).

**Figure 1:**
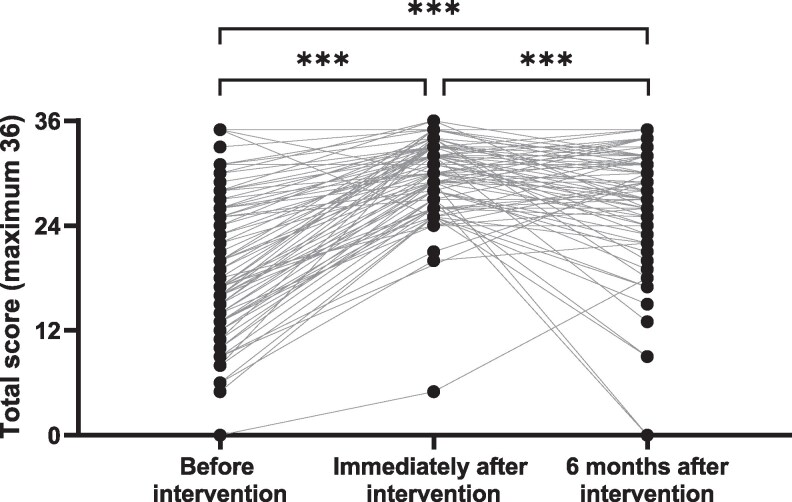
Scores increased significantly after the intervention compared to the baseline but declined at 6 months. Dots represent the IPC scores for a single healthcare facility at each time point. Lines represent paired observations from the same healthcare facility pre- and postintervention and at 6-month follow-up. Upward-sloping lines indicate improved IPC performance, and downward-sloping lines indicate deteriorating IPC performance. Total IPC scores ranged from 0 (lowest) to 36 (highest). Abbreviation: IPC, infection prevention and control. ***P < .0001.

At the follow-up survey, 6 months later, the total IPC score decreased to a median of 28 (IQR 24–32). This was statistically lower than the immediate postintervention score (*P* < .0001) but higher than the baseline (Figure 1, *P* < .0001). For 7 of the 36 items (19%) on the scorecard, the proportion of compliant healthcare facilities decreased significantly (Supplementary Table 1). Scorecard items that decreased included as follows: PPE is available to staff; staff wear appropriate PPE when handling waste; and cleaning staff wear appropriate PPE (Supplementary Table 1).

## DISCUSSION

Here we show that an IPC bundle intervention provided objective improvements in IPC scores at healthcare facilities during an EVD epidemic, but that the improvement was not fully sustained 6 months after the intervention.

A similar study examined the effect of a similar IPC bundle intervention during the 9th EVD outbreak in the DRC, with an increase in IPC score from 8% to 50% in hospitals and from 4% to 37% in health centers [9]. Other studies in Liberia [10] and Guinea [11] also documented improvements in IPC scores following training and supervision. Taken together, these data suggest that immediate gains in IPC measures can be achieved with a bundle intervention.

Five years after the large EVD epidemic in Liberia, IPC capacity was sustained, but tangible consumables were quickly depleted in the absence of sustained long-term investment by donors [12]. Similarly, in our study, 6 months after IPC donations ceased, the scorecard reflected a lack of PPE (Supplementary Table 1), highlighting the need to incorporate PPE into standard hospital procurement processes rather than reliance on external provision. On the other hand, a culture of IPC may have been at least partially retained, based on several scorecard items that remained higher than baseline 6 months after cessation of the intervention (Supplementary Table 1). Further longitudinal assessment is needed to better understand whether those initially sustained practices remain durable over time.

These findings have important implications for nosocomial transmission of EVD early in an outbreak, before reactive public health measures are implemented. The absence of sustained IPC practice exposes healthcare workers to potentially lethal EVD infection [13, 14] and facilitates early epidemic amplification within healthcare facilities [4, 5]. This pattern is exemplified by the recent Kasai outbreak, in which healthcare workers accounted for 4/45 of deaths [1]. Future measures to improve IPC durability may include institutionalization of IPC training within routine hospital practice, ongoing supervision by IPC focal persons, and incentive-based reinforcement [15].

This study has several limitations. Our quasi-experimental design is subject to confounding by changes over time other than the intervention. A randomized design would be needed to determine if a causal association exists between the IPC bundle intervention and the change in score. The Hawthorne effect may account for improvements in behavior-related IPC items. Ninety-two healthcare facilities were included from one geographic area. This sample may not be representative of all facilities in EVD endemic areas. Follow-up beyond 6 months postintervention and beyond the declaration of the end of the outbreak would be helpful to characterize longer-term temporal patterns of change across IPC domains. The IPC scorecard used was not formally validated; however, the components were based on existing Ebola IPC assessment frameworks and similar to those used in past studies [9]. Inter-rater reliability was not measured. Other clinically meaningful outcomes such as incidence of nosocomial transmission would be of interest for future studies but were beyond the scope of the current project.

In summary, our study provides confirmatory data that IPC capacity in resource-limited healthcare facilities is vulnerable to erosion in the absence of long-term investment [4]. Outbreak-prone, low-income countries need IPC measures not solely as an emergency response but as routine medical safety practice. Congolese healthcare facilities likely remain ill-equipped to safely manage highly transmissible EVD, despite past national experience. These results underscore the need to move beyond reactive measures toward a system-strengthening approach to sustain IPC programs and mitigate nosocomial EVD transmission in future epidemics.

## Supplementary Material

ciag192_Supplementary_Data

## Supplementary Data

Supplementary materials are available at *Clinical Infectious Diseases* online. Consisting of data provided by the authors to benefit the reader, the posted materials are not copyedited and are the sole responsibility of the authors, so questions or comments should be addressed to the corresponding author.
