# Supplementary material for: Limited Durability of Improvements in Infection Prevention and Control Practices Following Reactive Interventions Leaves Healthcare Facilities Vulnerable to Ebola Virus Transmission
Source: Clin Infect Dis. 2026 Mar 18;83(1):e130–2. doi: 10.1093/cid/ciag192 (PMC13393106; doi:10.1093/cid/ciag192)
Supplement: ciag192_Supplementary_Data [file ciag192_supplementary_data.docx]

**Supplemental Table. Changes in Infection Prevention and Control (IPC) practices before, immediately after, and six months after an IPC bundle intervention**

|  | **Item** | **Before intervention** | **Immediately after intervention** | **6 months after intervention** |
| --- | --- | --- | --- | --- |
| 1 | HCF has a focal point (FP, i.e., delegated contact person) or IPC committee with appropriate responsibility, accountability, and authority. | 71 (77) | 89 (97) | 81 (88) |
| 2 | Malaria rapid diagnostic tests (mRDTs) are available and the FP knows how to use them. | 43 (47) | 86 (93) | 75 (82) |
| 3 | PF has dedicated time allocated to perform his/her IPC tasks. | 46 (50) | 82 (89) | 71 (77) |
| 4 | Temperature and EVD symptoms are checked correctly – functional non-contact thermometer. | 69 (75) | 91 (99) | 69 (75)** |
| 5 | Triage sheet and register are available. | 59 (64) | 91 (99) | 68 (74)** |
| 6 | Correct use of the form and register. | 40 (43) | 83 (90) | 63 (68)** |
| 7 | Isolation area is well marked and is away from other units / services. | 39 (42) | 70 (76) | 69 (75) |
| 8 | Separate latrines / toilet are available in the isolation area, or a bedpan / urinal is available. | 29 (32) | 59 (64) | 62 (67) |
| 9 | Isolation area includes: a hand-washing station, supplies (PPE, a bed, bedpan / urinal, etc.); a zone to don PPE and a zone to doff PPE. | 33 (36) | 54 (59) | 57 (62) |
| 10 | Should include (clean water + soap and / or alcohol hand rub and / or 0.005% chlorine bleach (if the other two are not available). | 70 (76) | 86 (93) | 71 (77) |
| 11 | All staff are able to perform hand hygiene properly (following the WHO technique). | 26 (28) | 86 (93) | 74 (80) |
| 12 | Posters are displayed showing hand hygiene technique at every hand-washing station. | 69 (75) | 91 (99) | 81 (88) |
| 13 | PPE is accessible to staff at all times and in sufficient quantity in the dressing room. | 55 (60) | 78 (85) | 42 (46)** |
| 14 | Posters are displayed showing standard IPC precaution and EVD measures) on how to don and doff PPE. | 52 (57) | 85 (92) | 79 (86) |
| 15 | The staff able to don and doff PPE, correctly following all the steps. | 28 (30) | 77 (84) | 63 (68) |
| 16 | Trash bins are waterproof, covered, and labeled (infectious or non-infectious) and posters on waste management are displayed in all patient care areas. | 50 (54) | 86 (93) | 79 (86) |
| 17 | Sharps containers are available at all points where sharps are used. | 75 (82) | 89 (97) | 85 (92) |
| 18 | Waste is sorted according to type (e.g., indicated by color ): infectious, non-infectious, sharps) | 41 (45) | 81 (88) | 74 (80) |
| 19 | All staff wear appropriate PPE (latex or nitrile gloves, housekeeping gloves, protective eyewear, rubber boots, aprons, and masks) when handling waste. | 48 (52) | 83 (90) | 59 (64)** |
| 20 | Waste is incinerated on site in an incinerator, or a system is in place for transportation of waste to another appropriate location. | 78 (85) | 87 (95)* | 86 (93) |
| 21 | An organic waste pit is present, if required. | 43 (47) | 53 (58)* | 59 (64) |
| 22 | All staff have been trained at a minimum on universal standard precautions, additional precautions (practical and theoretical training) with an emphasis on EVD and/or hemorrhagic fevers in the past six months. | 31 (34) | 49 (53) | 53 (58) |
| 23 | A register is kept with the names of the HCWs who have undergone the training, with date, type of training and the organization that provided the training. | 36 (39) | 71 (77) | 73 (79) |
| 24 | HCWs receive ongoing training through on-site supervision. | 79 (86) | 89 (97)* | 63 (68)** |
| 25 | An Ebola alert number is known and is visibly displayed. | 86 (93) | 86 (93)* | 86 (93) |
| 26 | Hospitalized patients are screened at least twice a day to identify suspect cases. | 67 (73) | 85 (92) | 82 (89) |
| 27 | Once identified, suspect cases are moved to the isolation area or the transit area and an alert is activated. | 54 (59) | 84 (91) | 77 (84) |
| 28 | Sterilization equipment is available such as autoclave and accessories. | 56 (61) | 70 (76) | 55 (60) |
| 29 | An SOP is available on how to perform the sterilization of equipment and materials. | 24 (26) | 57 (62) | 64 (70) |
| 30 | Staff performing sterilization have been trained. | 17 (18) | 25 (27)* | 18 (20) |
| 31 | An SOP is available on how to clean/disinfect when there are body fluids or blood as well as cleaning and decontamination of reusable equipment. | 47 (51) | 82 (89) | 81 (88) |
| 32 | Staff performing cleaning and disinfection have been trained. | 44 (48) | 60 (65)* | 67 (73) |
| 33 | Cleaning staff wear appropriate PPE (latex or nitrile gloves, protective eyewear, rubber boots, aprons, and masks). | 43 (47) | 84 (91) | 62 (67)** |
| 34 | A protocol for assessment and management in case of EVD exposure is in place (including a register, evaluation tools, communication, etc.). | 38 (41) | 67 (73) | 68 (74) |
| 35 | The management of exposed HCWs is clearly defined and assured. | 55 (60) | 72 (78) | 73 (79) |
| 36 | The investigation team is alerted and mobilizes an investigation when a HCW is exposed. | 75 (82) | 81 (88)* | 79 (86) |

*Proportion of compliant sites was not statistically significantly different from baseline (before intervention) (p≥0.05/36)

** Proportion of compliant sites decreased significantly different from post-intervention survey (immediately after intervention) (p<0.05/36)
